# A Retrospective Multicenter Stratified Study on Perinatal Factors Influencing Neonatal Mortality in Preterm Infants in Kazakhstan

**DOI:** 10.1155/ijpe/8678975

**Published:** 2025-08-23

**Authors:** Nishankul Bozhbanbayeva, Olga An, Saltanat Sairankyzy, Indira Suleimenova, Aigul Bazarbayeva, Indira Adilbekova

**Affiliations:** ^1^Department of Neonatology, Asfendiyarov Kazakh National Medical University, Almaty, Kazakhstan; ^2^Department of Propaedeutics of Childhood Diseases, Asfendiyarov Kazakh National Medical University, Almaty, Kazakhstan; ^3^Department of Science and Postgraduate Education, Scientific Center of Pediatrics and Pediatric Surgery, Almaty, Kazakhstan

## Abstract

**Aim:** This study is aimed at evaluating the cumulative effect of postnatal risk factors on the survival of preterm neonates by examining key clinical parameters and complications across various gestational ages.

**Methods:** A retrospective cohort study was conducted using data from 1109 neonates admitted to neonatal intensive care units at two tertiary regional hospitals in Kazakhstan between 2021 and 2024. Patients were classified into three groups based on gestational age: extremely preterm (< 28 weeks, *n* = 223), very preterm (28–31 weeks, *n* = 384), and moderate to late preterm (32–36 weeks, *n* = 502). Initially, to identify significant risk factors, categorical variables were analyzed using the *χ*^2^ test or Fisher's exact test with the Bonferroni correction, depending on whether the expected counts were ≥ 5 or < 5, while continuous variables were examined with the Kruskal–Wallis and Mann–Whitney *U* tests. Subsequently, multivariate logistic regression was applied to develop a prognostic model for each study group based on previously identified statistically significant risk factors for neonatal mortality. The predictive performance of these factors was further evaluated through ROC curve analysis with AUC. Finally, the Kaplan-Meier method was used to reflect overall mortality outcomes, illustrating their association with gestational age and the distribution of fatal cases over time in days.

**Results:** The results of this study reveal significant differences in survival rates among preterm neonates based on gestational age, with mortality being most pronounced in infants born at less than 28 weeks of gestation. As gestational age increased among preterm infants, there was a marked reduction in the number of statistically significant independent risk factors identified in logistic models that influence survival. Disseminated intravascular coagulation consistently emerged as a significant predictor across all three logistic models. Neonatal depression, hyperbilirubinemia, and necrotizing enterocolitis were significant in both extremely preterm infants (less than 28 weeks) and very preterm infants (28–31 weeks). However, patent ductus arteriosus and bronchopulmonary dysplasia were statistically significant only in the extremely preterm group (less than 28 weeks).

**Conclusion:** In a cohort of preterm neonates in Kazakhstan, the cumulative impact of various risk factors plays a critical role in determining survival outcomes, with increasing gestational age significantly enhancing the likelihood of survival. Further research is required to refine prognostic models and identify factors specific to different sociogeographical populations.

## 1. Introduction

Prematurity is one of the leading causes of neonatal mortality and long-term morbidity, especially among infants with extremely low gestational age [1, 2]. Despite advances in perinatal medicine, the survival rates and outcomes for preterm newborns remain highly dependent on gestational age, birth weight, and the presence of postnatal complications [3–5]. Recent studies highlight the multifactorial nature of neonatal mortality, emphasizing the interplay between perinatal conditions, neonatal interventions, and the evolving capabilities of neonatal intensive care units (NICUs) [6, 7].

A growing body of research underscores the importance of identifying key predictors of neonatal mortality across different gestational age groups. Patent ductus arteriosus (PDA), bronchopulmonary dysplasia (BPD), hypoxic-ischemic encephalopathy (HIE), intraventricular hemorrhage (IVH), disseminated intravascular coagulation (DIC), necrotizing enterocolitis (NEC), and severe neonatal infections have been consistently associated with increased mortality risk among preterm infants [8–11]. Additionally, advancements in neonatal care, including early surfactant therapy, targeted temperature management, and improved respiratory support strategies, have significantly influenced survival patterns; yet disparities persist based on resource availability and regional healthcare policies [12–14].

These developments emphasize the need for region-specific investigations that account for variations in neonatal care protocols and population characteristics [15, 16].

This study is a retrospective analysis of preterm newborns admitted to the NICUs of two major regional tertiary hospitals in Kazakhstan.

The novelty of this study lies in the comprehensive evaluation of postnatal risk factors with prognostic value for survival across different degrees of prematurity within the regional setting of Kazakhstan. The findings may contribute to the improvement of management strategies for preterm infants and enhance the quality of medical care in specialized neonatal centers.

## 2. Materials and Methods

The study included 1109 preterm newborns born between 2021 and 2024 at two regional level III perinatal centers. These centers operate within the system of specialized neonatal care and provide treatment for all preterm newborns requiring intensive therapy. Preterm neonates with chromosomal anomalies, genetic disorders, or birth injuries that could significantly affect survival were excluded from the study. This exclusion specifically applied to birth injuries associated with moderate to severe birth asphyxia that result in significant clinical complications or require intensive resuscitation. Mild birth asphyxia was not considered an exclusion criterion. Thus, the sample is representative of the population of preterm newborns in the respective regions.

The statistical power of the *χ*^2^ test was calculated to assess the adequacy of the sample size. The analysis was conducted with a significance level of *α* = 0.05, an expected effect size (Cohen's *w*) of 0.3, and two degrees of freedom. With a total sample size of 1109, including group sizes of 223, 384, and 502, the calculated power of the test was 0.9999 or 99.99%. This result indicates a high ability to detect existing differences between groups and confirms that the sample size was sufficient for reliable statistical conclusions.

The stratification based on gestational age covers all key categories: < 28, 28–31, and 32–36 weeks' gestation, which allows for the extrapolation of the findings to the entire population of preterm infants in high-level hospitals, ensuring the reliability and generalizability of the statistical conclusions.

The study categorized preterm infants into three groups according to gestational age: Group 1—extremely preterm (< 28 weeks' gestation, n = 223); Group 2—very preterm (28–31 weeks' gestation, *n* = 384); and Group 3—moderate to late preterm (32–36 weeks' gestation, *n* = 502) [17].

The retrospective analysis was conducted based on data from medical documentation, which included demographic indicators, perinatal period parameters, characteristics of the early neonatal period, and the presence and severity of postnatal complications. Neonatal mortality served as the outcome variable, while key postnatal risk factors were considered as predictors.

Categorical data were analyzed using the chi-square (*χ*^2^) test, and in cases of statistical significance (*p* < 0.05), pairwise comparisons between the three observation groups were conducted with the Bonferroni correction (*α* = 0.0167). When contingency tables contained expected values less than 5, the extended Fisher's exact test (Freeman–Halton test, *α* = 0.0167) was applied. To ensure the validity of comparisons across groups with differing sample sizes, data normalization was performed.

The analysis of the distribution of IVH grades in preterm neonates by gestational age was conducted using the Kruskal–Wallis test. The choice of this nonparametric method was determined by the characteristics of the studied variable: IVH grade represents an ordinal scale reflecting the severity of the pathological process rather than independent categories. Additionally, the comparison groups varied significantly in size, and some categories exhibited zero or extremely low frequencies, violating the assumptions of the *χ*^2^ test. Under these conditions, the Kruskal–Wallis test provided a more reliable assessment of differences across three or more independent samples. For a more detailed pairwise comparison, the Mann–Whitney *U* test was applied, as it is more sensitive to the distribution of ranked data. Given the multiple comparisons, the Bonferroni correction was applied (*α* = 0.05/3 ≈ 0.0167).

To identify factors associated with the risk of mortality in preterm infants, a multivariate logistic regression analysis was performed, calculating odds ratios (OR) and 95% confidence intervals (CI). In addition to the regression analysis, a ROC analysis was conducted to assess the prognostic significance of factors influencing survival outcomes among preterm infants with varying gestational ages. The performance of the predictive model was evaluated using the area under the curve (AUC), which illustrates the relationship between sensitivity and specificity at different threshold values. This allows for an evaluation of the model's ability to accurately classify fatal and survival outcomes. The AUC is a critical measure of the model's accuracy, with higher AUC values indicating a better ability to discriminate between positive and negative outcomes.

The Kaplan–Meier method was used to assess the survival of preterm neonates, allowing for the analysis of survival time while accounting for censored data. A cumulative survival curve was constructed for three groups of preterm infants with different gestational ages. This approach provided a visual representation of survival dynamics across cohorts and enabled the identification of critical time intervals associated with the highest mortality rates.

The study was conducted in accordance with the principles of the Declaration of Helsinki and was approved by the Local Research Ethics Committee (LREC), as documented in Meeting Minutes No. 16, dated October 31, 2024.

## 3. Results

At the first stage of the study, statistically significant differences between the three groups were assessed based on key clinical indicators.  Table 1 summarizes the statistical calculations, including *χ*^2^ test values, Fisher's exact test, as well as the Kruskal–Wallis and Mann–Whitney tests. These results provide an assessment of differences between groups in terms of key clinical parameters such as neonatal mortality, disease severity, and survival, depending on gestational age and other factors.

The analysis revealed a progressive increase in survival rates with advancing gestational age, with neonates born at 32–36 weeks demonstrating significantly higher survival compared to those born at 28–31 weeks, while the latter had better outcomes than those born before 28 weeks. Statistically significant differences were observed across all three gestational age groups (*p* < 0.0001), emphasizing the pivotal role of gestational maturity in neonatal survival. In contrast, sex distribution did not show statistically significant differences (*p* = 0.073), indicating that sex does not play a significant role in determining the degree of prematurity in this neonatal cohort.

Apgar scores at the first and fifth minutes differed significantly across groups, with the lowest values in Group 1, indicating greater neonatal distress, and the highest in Group 3, reflecting better adaptation to extrauterine life. Statistically significant differences between Groups 2 and 3 further underscored the decisive role of gestational age in neonatal adaptation and outcomes (*p* < 0.0001).

Respiratory distress syndrome (RDS) was more frequently diagnosed in newborns with lower gestational age, with the most pronounced differences observed between Group 1 and the other groups (*p* < 0.0001). The frequency of acute neonatal respiratory failure was assessed using the extended Fisher's exact test, revealing that all neonates in Group 1 (< 28 weeks) experienced respiratory insufficiency (100%), underscoring their extreme vulnerability to pulmonary complications. In Group 2 (28–31 weeks), respiratory failure remained prevalent at 98.4%, though the difference from Group 1 was not statistically significant (*p* = 0.2713), suggesting comparable severity in extremely and very preterm infants born before 32 weeks. In contrast, among moderate to late preterm newborns (32–36 weeks), respiratory impairment was diagnosed in 87.3% of cases, a significantly lower proportion than in the first two groups (*p* = 7.55 × 10^−11^; *p* = 1.49 × 10^−10^), reflecting a progressive reduction in the risk of pulmonary complications with increasing gestational age. Similarly, BPD was notably more prevalent in Group 1, where all newborns had respiratory failure, whereas in Group 3, it was identified in only 4 out of 502 cases (0.8%), a markedly lower rate than in the first two groups (0.00025). This evidence points to the crucial role of lung maturity in the pathogenesis of neonatal respiratory disorders and highlights the necessity of timely and intensive respiratory support for the most preterm infants, who are at the greatest risk of severe pulmonary complication.

Transient tachypnea of the newborn (TTN) was significantly more common in infants with a gestational age of 32–36 weeks compared to those born earlier (*p* < 0.0001). No significant differences were observed between extremely and very preterm neonates born before 32 weeks of gestation (*p* = 0.558), suggesting distinct transitional respiratory adaptation patterns in moderate to late preterm infants. TTN results from delayed resorption of fetal lung fluid [18]. Notably, its lower incidence in infants with earlier gestational ages is likely due to the predominance of surfactant deficiency-related RDS and the increased use of respiratory support, which facilitates fluid clearance. These findings underscore the substantial differences in respiratory adaptation mechanisms across gestational ages and highlight the need for individualized respiratory management strategies in moderate to late preterm neonates.

PDA was significantly more common in neonates with a gestational age of less than 28 weeks compared to those born at 32–36 weeks (*p* < 0.0001). No differences were found between infants born before 28 weeks and those born at 28–31 weeks (*p* = 1.0), confirming the higher prevalence of PDA among the most immature neonates. According to the *χ*^2^ analysis, no significant differences in the frequency of atrial septal defect (ASD) were found between the groups (*p* = 0.0813). These observations illustrate that gestational age does not have a significant impact on the incidence of ASD in preterm infants within this cohort.

Neonatal HIE was significantly more common in infants born before 32 weeks of gestation (*p* < 0.0001), with no differences observed between the < 28-week and 28–31-week groups (*p* = 0.3386), indicating a decreasing risk of HIE with increasing gestational age. A similar pattern was observed for non-traumatic IVH, with its incidence and severity inversely correlated with gestational age (*H* = 211.64, *p* < 0.0001). This association can be attributed to the high vascular fragility of the germinal matrix and the heightened susceptibility of the most immature neonates to hypoxic-ischemic injury. The distribution of IVH grades across different gestational age groups was analyzed using the Kruskal–Wallis test, which was selected due to the ordinal nature of the variable, reflecting the severity of the pathological process rather than independent categorical variables. For pairwise comparisons, the Mann–Whitney *U* test was applied. The analysis revealed that mild IVH (Grade I) was significantly more common in the more mature group (32–36 weeks) at 37%, compared to 18.4% in the extremely preterm group (< 28 weeks). In contrast, severe IVH (Grade IV) was observed exclusively in the < 28-week group, with an incidence of 1.3%, and was absent in the 32–36-week group. Despite the peak prevalence of neonatal depression in the 28–31-week group, which was significantly higher than in the < 28-week and 32–36-week groups, further pairwise comparisons with Bonferroni correction revealed statistically significant differences between Groups 1 and 2 (*p* < 0.0001) and between Groups 2 and 3 (*p* < 0.0002), overall confirming the inverse relationship between increasing gestational age and the incidence of neonatal depression. Neonatal seizures were significantly more frequent in newborns with a gestational age of < 28 weeks (*p* < 0.0001), whereas their incidence was substantially lower in the 28–31-week and 32–36-week groups, with no significant differences between them (*p* = 0.224), confirming the highest risk of seizures in extremely preterm infants.

Hemorrhagic disease of the newborn (HDN), DIC, and preterm anemia exhibited a strong dependence on gestational age (*p* < 0.0001), reflecting the degree of hemostatic and vascular immaturity. These pathological conditions were most frequently observed in neonates with a gestational age of < 28 weeks, whereas their incidence was significantly lower in the 32–36-week group, with statistically significant differences identified among all three groups (*p* < 0.01).

Intrauterine pneumonia was more prevalent among neonates with lower gestational age (*p* < 0.0001), with particularly pronounced differences observed between Groups 2 and 3. However, no significant differences were found between Groups 1 and 2 (*p* = 0.109), confirming a reduction in the risk of this factor with increasing gestational age. Similarly, NEC was significantly more frequent in neonates born at < 28 weeks (26.5%), with a marked decrease in incidence in the 32–36-week group (1%), as evidenced by statistically significant differences across all groups. A similar trend was observed for TORCH infections, which were most prevalent in the <28-week group (17.9%) and decreased with advancing gestational age, reaching a minimum in the 32–36-week group (1.8%). Neonatal hyperbilirubinemia was more common in infants born at < 28 weeks (56.9%) and 28–31 weeks (63.8%) compared to those born at 32–36 weeks (33.3%). No significant differences were found between the <28-week and 28–31-week groups (*p* = 0.339).

At the second stage of the study, a multivariate logistic regression model was calculated separately for each of the three groups to assess the association between factors that showed significant differences in the comparative analysis and neonatal survival outcomes; followed by the construction of the ROC curve and the calculation of the AUC to evaluate the predictive performance of the identified factors.

The following independent determinant factors were included in the regression analysis to assess their association with mortality: gestational age, Apgar scores at the 1st and 5th minutes, RDS, BPD, TTN, PDA, IVH of varying severity, neonatal pneumonia, HIE, neonatal depression, HDN, neonatal hyperbilirubinemia, DIC, AOP, NEC, TORCH infections (toxoplasmosis, other infections, rubella, cytomegalovirus, herpes), and neonatal seizures. Neonatal mortality was considered the dependent variable.

Presented below is the multivariate logistic regression model for extremely preterm infants born before 28 weeks of gestation, evaluating the impact of perinatal and neonatal factors on mortality in this high-risk group (Table 2).

The results indicated that the overall model was statistically significant *χ*^2^(18) = 237.2, *p* < 0.001, *n* = 223, demonstrating a strong association between the independent variables and the dependent variable.

The key predictors of neonatal mortality were identified as PDA, neonatal depression, neonatal jaundice, DIC, and NEC, exerting the most significant influence on survival outcomes. PDA was statistically significant (*p* = 0.003) with an OR of 0.05 (95% CI: 0.01–0.35), indicating a strong association with mortality. Similarly, neonatal depression (*p* < 0.001, OR = 0.01, 95% CI: 0–0.12) and neonatal jaundice (*p* = 0.005, OR = 0.05, 95% CI: 0.01–0.4) showed a substantial impact. DIC (*p* = 0.002, OR = 35.51, 95% CI: 3.76–335.18) and NEC (*p* < 0.001, OR = 59.76, 95% CI: 7.29–489.69) were also critical determinants of mortality risk in this cohort of preterm infants.

Gestational age (*p* = 0.013, OR = 0.41, 95% CI: 0.2–0.82) and BPD (*p* = 0.012, OR = 0.01, 95% CI: 0–0.35) had a moderate impact on mortality but were less influential compared to the primary predictors.

In contrast, factors such as Apgar scores, RDS, TTN, IVH, congenital pneumonia, HIE, hemorrhagic disease, AOP, TORCH infections, and neonatal seizures did not demonstrate a statistically significant association with mortality. These variables had *p*-values above 0.05, indicating insufficient correlation with fatal outcomes in this cohort.

**Figure 1:**
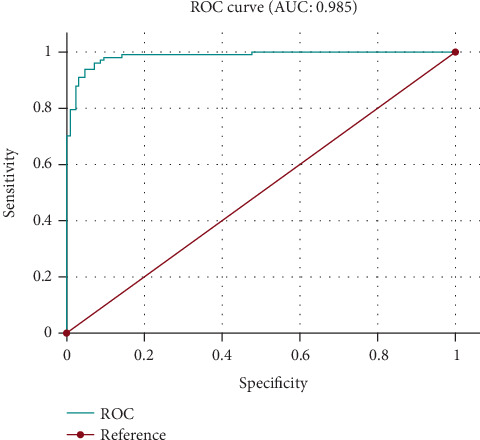
ROC curve illustrating the predictive value of factors influencing survival in preterm infants born before 28 weeks of gestation.

Below is the ROC curve for the first logistic regression model predicting mortality in extremely preterm infants (Figure 1). In this case, the ROC curve with an AUC of 0.985 indicates a high degree of accuracy in predicting neonatal mortality among extremely preterm infants. While this AUC value suggests strong discrimination between survivors and fatalities, it is important to emphasize that these findings are specific to this cohort. The model's performance may vary in different populations or settings; further research is needed to assess its reliability across broader contexts.

A logistic regression model was similarly constructed for the second group of preterm infants (Table 3). The analysis examined the impact of various neonatal risk factors on the likelihood of mortality, with the dependent variable representing the outcome (survived or not survived). A total of 384 cases were included in the study, and the overall model quality was statistically significant *χ*^2^(18) = 155.03, *p* < 0.001.

In the regression analysis of the second group of observations, several statistically significant factors influencing mortality among very preterm neonates (28–31 weeks gestation) were identified. The most significant factors influencing mortality were neonatal depression (*p* < 0.001, OR = 15.12, 95% CI: 4.52–50.61) and DIC (*p* < 0.001, OR = 0.07, 95% CI: 0.02–0.26), with particularly high impact on mortality risk. A less significant factor was neonatal hyperbilirubinemia (*p* = 0.002, OR = 6.31, 95% CI: 1.91–20.77), which increased the likelihood of fatal outcomes. The least significant factor was NEC, which, despite reducing the risk of death, was less influential (*p* = 0.046, OR = 0.22, 95% CI: 0.05–0.97).

Among all the factors considered in the analysis, neonatal depression emerged as the strongest predictor of mortality, with a coefficient of *b* = 2.72 (*p* < 0.001), increasing the likelihood of an adverse outcome by 15.12 times. Other factors, such as HIE, neonatal hyperbilirubinemia, and other complications, also demonstrated some significance, although their impact was less pronounced. For instance, HIE increased the probability of mortality by 3.02 times but did not reach statistical significance (*p* = 0.052).

**Figure 2:**
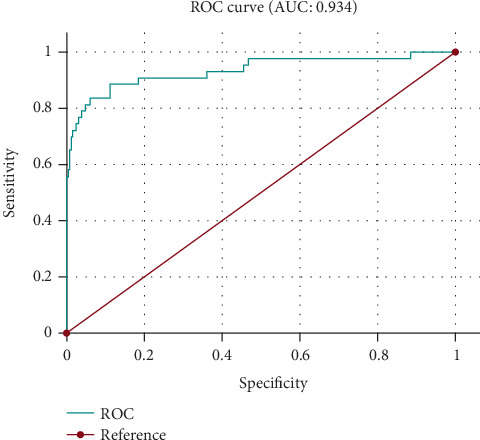
ROC curve illustrating the predictive value of factors influencing survival in preterm infants born between 28 and 31 weeks of gestation.

The ROC curve below illustrates the performance of the second logistic regression model for predicting mortality in very preterm neonates (Figure 2).

An AUC of 0.934 reflects the mode's high accuracy, emphasizing its robust ability to predict mortality based on the analyzed risk factors in extremely preterm neonates at 28–31 weeks' gestation.

Table 4 presents the third model investigating predictors of neonatal mortality among moderate to late preterm neonates (32–36 weeks gestation), based on the analysis of 502 cases, demonstrating statistically significant results (*χ*^2^(18) = 47.53, *p* < 0.001).

Among all the factors analyzed, DIC emerged as the only statistically significant predictor of neonatal mortality. This factor demonstrated a substantial impact on the risk of fatal outcomes, with an OR of 43,151.59 and a 95% CI (1.77–1,054,626,464.67, *p* = 0.038).

In contrast, most other predictors, such as BPD, IVH, neonatal depression, HDN, AOP, NEC, TORCH infections, and neonatal seizures, in this cohort of preterm newborns (32-36 weeks gestation), showed a CI of 0 to ∞, suggesting that these factors did not demonstrate any statistically significant associations with neonatal mortality.

In the group of moderate to late preterm infants (32–36 weeks gestation), particular attention should be given to predictors such as congenital pneumonia, IVH, neonatal depression, HDN, AOP, NEC, TORCH infections, and neonatal seizures. These factors demonstrated extremely high ORs; however, their CIs were excessively wide or included infinity, which may indicate model instability, issues with multicollinearity, and sample size limitations, as only 6 neonatal deaths were recorded out of 502 cases. To enhance model reliability and improve statistical significance, further research with an increased sample size is recommended, which will strengthen statistical power and improve predictive accuracy.

**Figure 3:**
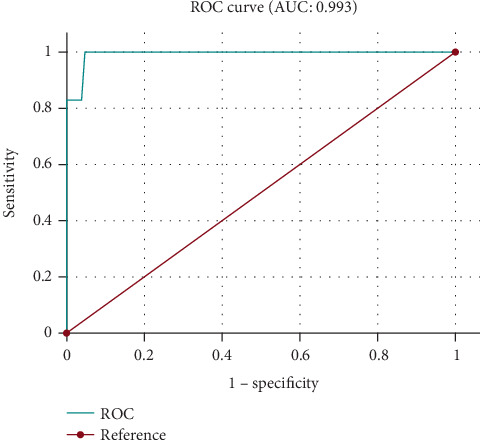
ROC curve illustrating the predictive value of factors influencing survival in preterm infants born between 32 and 36 weeks of gestation.

The ROC curve below illustrates the performance of the third logistic regression model in predicting mortality among moderate to late preterm neonates of 32–36 weeks' gestation (Figure 3).

Despite the almost perfect discriminatory ability of the predictive model (AUC = 0.993) in distinguishing between survivors and nonsurvivors in the moderate to late preterm group, the extreme class imbalance (only 6 fatal cases out of 502) raises concerns about its statistical stability and generalizability. This limits the model's ability to accurately predict neonatal mortality in broader clinical settings, while the wide CIs of the AUC in this context indicate potential variability in predictive accuracy. Therefore, to confirm the model's reliability, external validation on a larger and more balanced sample is necessary to ensure its applicability in clinical practice.

**Figure 4:**
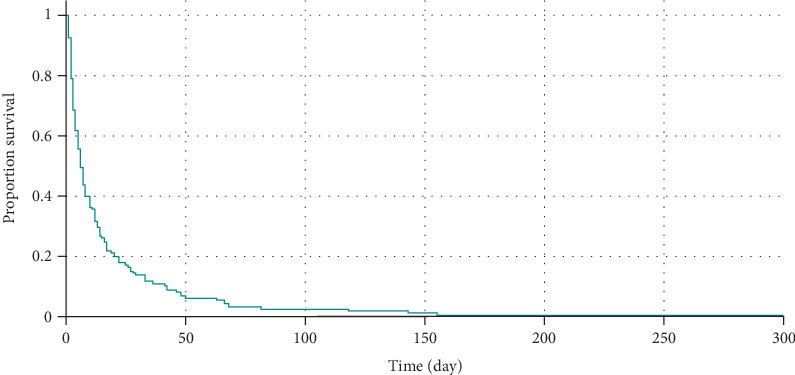
Kaplan–Meier survival analysis of preterm neonates stratified by gestational age.

At the final stage of the study, an overall Kaplan–Meier curve was constructed for all three groups of preterm neonates, revealing a clear relationship between gestational age and survival: the lower the gestational age, the higher the mortality rate (Figure 4). The curve demonstrated a pronounced decline in survival as gestational age decreased, confirming its significance as a key predictor of neonatal outcomes. These findings underscore the critical role of gestational age in survival prediction, highlighting the need for special consideration when making treatment and care decisions for preterm infants.

## 4. Discussion

The results of this study demonstrate notable variations in survival rates among preterm neonates based on gestational age. Mortality was found to be most pronounced in infants born at less than 28 weeks of gestation, corroborating global data that identifies this group as having the highest risk of mortality [9, 10, 19]. Extreme prematurity is typically associated with profound organ and system immaturity, making these infants particularly vulnerable [20–22]. The data from this study indicate that among more mature groups, such as very preterm (28–31 weeks) and moderate to late preterm (32–36 weeks) infants, reduced survival was also observed, though to a lesser extent.

As the gestational age increases among preterm infants, there is a noticeable decrease in the number of statistically significant independent risk factors identified in logistic models that affect survival. DIC was consistently observed as a significant predictor across all three logistic models. Neonatal depression, hyperbilirubinemia, and NEC were significant in the groups of extremely preterm infants (less than 28 weeks) and very preterm infants (28–31 weeks), whereas PDA and BPD were statistically significant only in the group of extremely preterm infants (less than 28 weeks).

These findings align with previous research highlighting the impact of these complications on survival outcomes in this gestational age range. For instance, a study on neonatal DIC emphasized its association with high mortality and severe complications, underscoring the critical nature of this condition in preterm infants [11].

A high predictive potential was identified for several risk factors, including neonatal depression and NEC, particularly among extremely preterm infants (< 28 weeks). These factors emerged as key predictors of mortality, aligning with global studies that identify NEC as a leading cause of death in this population. Ginglen and Butki emphasized its severe complications and clinical significance, reporting mortality rates of up to 50% and highlighting NEC as a major contributor to neonatal mortality [23].

Recent studies have highlighted the interplay between NEC and hypoxic-ischemic insults. For instance, research indicates that hypoxic-ischemic events can predispose near-term infants with congenital heart disease to NEC, suggesting a shared pathophysiological pathway [24]. This association underscores the vulnerability of preterm infants to multiple simultaneous insults, exacerbating their overall prognosis. The co-occurrence of NEC and HIE presents a compounded risk, leading to a significant increase in neonatal mortality. Infants suffering from both conditions are more likely to experience adverse outcomes, including severe neurodevelopmental impairments and prolonged hospitalizations. The combined impact of these conditions necessitates a comprehensive approach to neonatal care, emphasizing early detection and intervention strategies. Understanding the synergistic effect of NEC and hypoxic-ischemic conditions helps in developing targeted therapeutic interventions. Ongoing research aims to elucidate the mechanisms linking these pathologies, with the goal of improving prevention and treatment protocols.

The findings of this study align with global trends, reaffirming that gestational age remains a key determinant of survival in preterm neonates [25]. Identifying major predictors of mortality enables the development of targeted early interventions and preventive strategies, which can improve outcomes and optimize resource allocation in NICs. To effectively implement this approach, further global and regional studies are required, taking into account demographic, socioeconomic, and medical factors influencing preterm infant survival.

## 5. Limitations

The main limitation of this study is its retrospective design, which precludes establishing causal relationships between risk factors and survival outcomes. Additionally, the limited number of fatal cases (only 6 out of 502) in Group 3 with a gestational age of 32–36 weeks raises concerns about the statistical stability and generalizability of the model, which limits its ability to accurately predict neonatal mortality in broader clinical settings. To confirm the model's reliability, external validation on a larger and more balanced sample is required to ensure its applicability in clinical practice.

## 6. Conclusion

This study demonstrates that preterm neonatal survival is primarily influenced by gestational age and the combined effect of multiple risk factors. Mortality rates were highest among extremely preterm infants, highlighting their increased vulnerability. Early identification of risk factors and timely management of critical conditions are essential for improving outcomes and reducing neonatal mortality. Further research in diverse sociogeographical settings is needed to refine prognostic models, optimize neonatal care strategies, and identify population-specific determinants of survival.

## Figures and Tables

**Table 1:** Results of statistical calculations for differences between groups based on key clinical indicators.

**No.**	**Indicators**	**Group 1** **(< 28 weeks)**	**Group 2** **(28–31 weeks)**	**Group 3** **(32–36 weeks)**	**Statistical analysis results**
		**n**_1_ = 223	**n**_2_ = 384	**n**_3_ = 502	
1	Outcome				*χ* ^2^ = 243.69, df = 2, *p* < 0.0001 n₁ vs. n₂: *χ*^2^ = 81.13, *p* < 0.0001 n₁ vs. n₃: *χ*^2^ = 223.24, *p* < 0.0001 n₂ vs. n₃: *χ*^2^ = 39.77, *p* < 0.0001
	Survivor	126 (56, 5%)	341 (88, 8%)	496 (98, 8%)	
	Nonsurvivor	97 (43, 5%)	43 (11, 2%)	6 (1, 2%)	

2	Sex				*χ* ^2^ = 5.23, df = 2, *p* = 0.073
	Male	124 (55, 6%)	194 (50, 5%)	239 (47, 6%)	
	Female	99 (44, 4%)	190 (49, 5%)	263 (52, 4%)	

3	Apgar scores at the 1st minute				*χ* ^2^ = 512.34, df = 16, *p* < 0.0001 n₁ vs. n₂: *χ*^2^ = 198.67, *p* < 0.0001 n₁ vs. n₃: *χ*^2^ = 435.92, *p* < 0.0001 n₂ vs. n₃: *χ*^2^ = 172.14, *p* < 0.0001
	0	—	—	—	
	1	71 (31, 8%)	10 (2, 6%)	3 (0, 6%)	
	2	57 (25, 6%)	33 (8, 6%)	4 (0, 8%)	
	3	47 (21, 1%)	34 (8, 9%)	9 (1, 8%)	
	4	31 (13, 9%)	114 (29, 7%)	30 (5, 9%)	
	5	16 (7, 2%)	112 (29, 1%)	106 (21, 1%)	
	6	1 (0, 4%)	78 (20, 3%)	277 (55, 2%)	
	7	—	1 (0, 3%)	62 (12, 4%)	
	8	—	2 (0, 5%)	8 (1, 6%)	
	9	—	—	3 (0, 6%)	
	10	—	—	—	

4	Apgar at the 5th minutes				*χ* ^2^ = 687.54, df = 16, *p* < 0.0001 n₁ vs. n₂: *χ*^2^ = 275.39, *p* < 0.0001 n₁ vs. n₃: *χ*^2^ = 528.71, *p* < 0.0001 n₂ vs. n₃: *χ*^2^ = 191.03, *p* < 0.0001
	0	—	—	—	
	1	1 (0, 4%)	—	—	
	2	16 (7, 2%)	4 (1, 0%)	3 (%)	
	3	58 (26, 0%)	11 (2, 9%)	1 (%)	
	4	63 (28, 3%)	27 (7, 0%)	3 (%)	
	5	44 (19, 7%)	45 (11, 7%)	16 (%)	
	6	27 (12, 1%)	137 (35, 7%)	46 (%)	
	7	14 (6, 3%)	157 (40, 9%)	348 (%)	
	8	—	1 (0, 3%)	73 (%)	
	9	—	2 (0, 5%)	10 (%)	
	10	—	—	2 (%)	

5	Respiratory distress syndrome (RDS)				*χ* ^2^ = 71.39, df = 2, *p* < 0.0001. n₁ vs. n₂: *χ*^2^ = 5.91, *p* = 0.015 n₁ vs. n₃: *χ*^2^ = 60.36, *p* < 0.0001 n₂ vs. n₃: *χ*^2^ = 10.64, *p* = 0.0011
	Absent	44 (19, 7%)	121 (31, 5%)	253 (50, 4%)	
	Present	179 (80, 3%)	263 (68, 5%)	249 (49, 6%)	

6	Acute neonatal respiratory failure				n₁ vs. n₂: p = 0.2713 n₁ vs. n₃: *p* = 7.55 × 10^−11^ n₂ vs. n₃: *p* = 1.49 × 10^−10^
	Absent	0 (%)	6 (1, 6%)	64 (12, 7%)	
	Present	223 (100%)	378 (98, 4%)	438 (87, 3%)	

7	Bronchopulmonary Dysplasia (BPD)				n₁ vs. n₂: *p* = 0.00025 n₁ vs. n₃: *p* < 0.0000000000014 n₂ vs. n₃: *p* = 0.00028
	Absent	193 (86, 5%)	366 (95, 3%)	498 (99, 2%)	
	Present	30 (13, 5%)	18 (4, 7%)	4 (0, 8%)	

8	Transient tachypnea of the newborn (TTN)				n₁ vs. n₂: *p* = 0.558 n₁ vs. n₃: *p* < 0.0001 n₂ vs. n₃: *p* < 0.0001
	Absent	221 (99, 1%)	383 (99, 7%)	387 (77, 1%)	
	Present	2 (0, 9%)	1 (0, 3%)	115 (22, 9%)	

9	Patent ductus arteriosus (PDA)				*χ* ^2^ = 54.30, df = 2, *p* < 0.0001. n₁ vs. n₂: *χ*^2^ = 0.16, *p* = 1.0 n₁ vs. n₃: *χ*^2^ = 30.91, *p* < 0.0001 n₂ vs. n₃: *χ*^2^ = 37.83, *p* < 0.0001
	Absent	50 (22, 4%)	93 (24, 2%)	223 (44, 4%)	
	Present	173 (77, 6%)	291 (75, 8%)	279 (55, 6%)	

10	Atrial septal defect (ASD)				*χ* ^2^ = 5.02, df = 2, *p* = 0.0813
	Absent	201 (90, 1%)	346 (90, 1%)	471 (93, 8%)	
	Present	22 (9, 9%)	38 (9, 9%)	31 (6, 2%)	

11	Intraventricular hemorrhage (IVH) (per J. Volpe):				^a^ *H* = 211.64, *p* < 0.0001 n₁ vs. n₂: *p* = 0.012 n₁ vs. n₃: *p* < 0.0001 n₂ vs. n₃: *p* < 0.0001
	Grade I	41 (18, 4%)	135 (35, 2%)	186 (37%)	
	Grade II	94 (42, 2%)	126 (32, 8%)	27 (5, 4%)	
	Grade III	32 (14, 3%)	10 (2, 6%)	0 (0%)	
	Grade IV	3 (1, 3%)	2 (0, 5%)	0 (0%)	
	Absent	53 (23, 8%)	111 (28, 9%)	289 (57, 6%)	

12	Neonatal pneumonia				*χ* ^2^ = 362.29, df = 2, *p* < 0.0001 n₁ vs. n₂: *χ*^2^ = 4.38, *p* = 0.109 n₁ vs. n₃: *χ*^2^ ≈ 343.1, *p* < 0.0001 n₂ vs. n₃: *χ*^2^ ≈ 360.0, *p* < 0.0001
	Absent	29 (13%)	77 (20,1%)	372 (74,1%)	
	Present	194 (87%)	307 (79,9%)	130 (25,9%)	

13	Hypoxic-ischemic encephalopathy (HIE)				*χ* ^2^ = 99.02, df = 2, *p* < 0.0001
	Absent	126 (56, 5%)	190 (49, 5%)	403 (80, 3%)	n₁ vs. n₂: *χ*^2^ = 2.51, *p* = 0.3386
	Present	97 (43, 5%)	194 (50, 5%)	99 (19, 7%)	n₁ vs. n₃: *χ*^2^ = 43.06, *p* < 0.0001 n₂ vs. n₃: *χ*^2^ = 91.86, *p* < 0.0001

14	Neonatal depression				*χ* ^2^ = 31.64, df = 2, *p* < 0.0001
	Absent	92 (41, 3%)	79 (20, 6%)	166 (33, 1%)	n₁ vs. n₂: *χ*^2^ = 28.81, *p* < 0.0001
	Present	131 (58, 7%)	305 (79, 4%)	336 (66, 9%)	n₁ vs. n₃: *χ*^2^ = 4.17, *p* = 0.124 n₂ vs. n₃: *χ*^2^ = 16.36, *p* < 0.0002

15	Hemorrhagic disease of newborn (HDN)				*χ* ^2^ = 266.23, df = 2, *p* < 0.0001
	Absent	70 (31, 4%)	176 (45, 8%)	438 (87, 3%)	n₁ vs. n₂: *χ*^2^ = 11.62, *p* = 0.0020
	Present	153 (68, 6%)	208 (54, 2%)	64 (12, 7%)	n₁ vs. n₃: *χ*^2^ = 227.09, *p* < 0.0001 n₂ vs. n₃: *χ*^2^ = 173.49, *p* < 0.0001

16	Neonatal hyperbilirubinemia				*χ* ^2^ = 88.99, df = 2, *p* < 0.0001 n₁ vs. n₂: *χ*^2^ = 2.51, *p* = 0.339 n₁ vs. n₃: *χ*^2^ = 34.95, *p* < 0.0001 n₂ vs. n₃: *χ*^2^ = 80.32, *p* < 0.0001
	Absent	96 (43, 1%)	139 (36, 2%)	335 (66, 7%)	
	Present	127 (56, 9%)	245 (63, 8%)	167 (33, 3%)	

17	Disseminated intravascular coagulation (DIC)				*χ* ^2^ = 306.40, df = 2, *p* < 0.0001 n₁ vs. n₂: *χ*^2^ = 70.70, *p* < 0.0001 n₁ vs. n₃: *χ*^2^ = 316.85, *p* < 0.0001 n₂ vs. n₃: *χ*^2^ = 105.38, *p* < 0.0001
	Absent	76 (34, 1%)	267 (69, 5%)	478 (95, 2%)	
	Present	147 (65, 9%)	117 (30, 5%)	24 (4, 8%)	

18	Anemia of prematurity (AOP)				*χ* ^2^ = 265.70, df = 2, *p* < 0.0001 n₁ vs. n₂: *p* < 0.0001 n₁ vs. n₃: *p* < 0.0001 n₂ vs. n₃: *p* < 0.0001
	Absent	102 (45, 7%)	285 (74, 2%)	492 (98%)	
	Present	121 (54, 3%)	99 (25, 8%)	10 (2%)	

19	Necrotizing enterocolitis (NEC)				*χ* ^2^ = 117.35, df = 2, *p* < 0.0001 n₁ vs. n₂: *p* < 0.0001 n₁ vs. n₃: *p* < 0.0001 n₂ vs. n₃: *p* = 0.0002
	Absent	164 (73, 5%)	355 (92, 4%)	497 (99%)	
	Present	59 (26, 5%)	29 (7, 6%)	5 (1%)	

20	TORCH infections				*χ* ^2^ = 60.73, df = 2, *p* < 0.0001 n₁ vs. n₂: *p* = 0.0003 n₁ vs. n₃: *p* < 0.0001 n₂ vs. n₃: *p* = 0.0041
	Absent	183 (82, 1%)	355 (92, 4%)	493 (98, 2%)	
	Present	40 (17, 9%)	29 (7, 6%)	9 (1, 8%)	

21	Neonatal seizures				n₁ vs. n₂: *p* < 0.0001 n₁ vs. n₃: *p* < 0.0001 n₂ vs. n₃: *p* = 0.224
	Absent	73 (32, 7%)	377 (98, 2%)	498 (99, 2%)	
	Present	150 (67, 3%)	7 (1, 8%)	4 (0, 8%)	

^a^ Kruskal–Wallis test and pairwise comparisons using the Mann–Whitney test with Bonferroni correction (*α* = 0.05/3 ≈ 0.0167):

**Table 2:** Evaluation of neonatal factors influencing survival in preterm infants born before 28 weeks of gestation.

	**Coefficient B**	**Standard error**	**z**	**p**	**Odds ratio**	**95% conf. interval**
Constant	27.39	9.33	2.94	0.003	785308492965.11	9011.61–68434977510132060000
Gestational age	−0.9	0.36	2.49	0.013	0.41	0.2–0.82
Apgar scores at the 1st minute	−1.06	0.92	1.15	0.251	0.35	0.06–2.12
Apgar scores at the 5th minutes	0.61	0.88	0.69	0.489	1.83	0.33–10.23
Respiratory distress syndrome (RDS)	1.33	1.07	1.24	0.213	3.78	0.47–30.67
Bronchopulmonary dysplasia (BPD)	−4.79	1.91	2.51	0.012	0.01	0–0.35
Transient tachypnea of the newborn (TTN)	−16.09	6818.56	0	0.998	0	0–∞
Patent ductus arteriosus (PDA)	−3.04	1.01	3	0.003	0.05	0.01–0.35
Intraventricular hemorrhage (IVH)	0.43	0.35	1.22	0.223	1.54	0.77–3.09
Neonatal pneumonia	−1.63	1.44	1.13	0.257	0.2	0.01–3.3
Hypoxic-ischemic encephalopathy (HIE)	−1.3	0.97	1.34	0.179	0.27	0.04–1.82
Neonatal depression	−4.26	1.11	3.85	< 0.001	0.01	0–0.12
Hemorrhagic disease of newborn (HDN)	−2.72	2.02	1.35	0.178	0.07	0–3.44
Neonatal hyperbilirubinemia	−3.02	1.07	2.83	0.005	0.05	0.01–0.4
Disseminated intravascular coagulation (DIC)	3.57	1.15	3.12	0.002	35.51	3.76–335.18
Anemia of prematurity (AOP)	0.18	0.83	0.22	0.83	1.2	0.23–6.15
Necrotizing enterocolitis (NEC)	4.09	1.07	3.81	< 0.001	59.76	7.29–489.69
TORCH infections	0.39	0.96	0.4	0.689	1.47	0.22–9.75
Neonatal seizures	2.46	2.09	1.18	0.239	11.75	0.19–708.7

**Table 3:** Evaluation of neonatal factors influencing survival in preterm infants born between 28 and 31 weeks of gestation.

	**Coefficient B**	**Standard error**	**z**	**p**	**Odds ratio**	**95% conf. interval**
Constant	8.56	8.05	1.06	0.287	5227.09	0–36982543852.19
Gestational age	−0.26	0.27	0.98	0.328	0.77	0.46–1.3
Apgar scores at the 1st minute	0.08	0.55	0.15	0.879	1.09	0.37–3.17
Apgar scores at the 5th minutes	−0.62	0.59	1.04	0.299	0.54	0.17–1.73
Respiratory distress syndrome (RDS)	−0.6	0.65	0.92	0.356	0.55	0.15–1.96
Bronchopulmonary dysplasia (BPD)	−0.91	1.2	0.76	0.449	0.4	0.04–4.23
Transient tachypnea of the newborn (TTN)	−16.49	19099.07	0	0.999	0	0–∞
Patent ductus arteriosus (PDA)	0.33	0.79	0.42	0.672	1.4	0.3–6.58
Intraventricular hemorrhage (IVH)	0.35	0.32	1.12	0.264	1.42	0.77–2.64
Neonatal pneumonia	−0.21	0.89	0.24	0.814	0.81	0.14–4.67
Hypoxic-ischemic encephalopathy (HIE)	1.11	0.57	1.94	0.052	3.02	0.99–9.25
Neonatal depression	2.72	0.62	4.41	< 0.001	15.12	4.52–50.61
Hemorrhagic disease of newborn (HDN)	−0.53	0.63	0.84	0.401	0.59	0.17–2.03
Neonatal hyperbilirubinemia	1.84	0.61	3.03	0.002	6.31	1.91–20.77
Disseminated intravascular coagulation (DIC)	−2.64	0.65	4.05	< 0.001	0.07	0.02–0.26
Anemia of prematurity (AOP)	0.29	0.64	0.45	0.653	1.33	0.38–4.64
Necrotizing enterocolitis (NEC)	−1.49	0.75	2	0.046	0.22	0.05–0.97
TORCH infections	0.59	0.95	0.62	0.535	1.8	0.28–11.49
Neonatal seizures	−19.63	6955.9	0	0.998	0	0–∞

**Table 4:** Evaluation of neonatal factors influencing survival in preterm infants born between 32 and 36 weeks of gestation.

	**Coefficient B**	**Standard error**	**z**	**p**	**Odds ratio**	**95% conf. interval**
Constant	−24.86	26891.13	0	0.999	0	0–∞
Gestational age	−0.88	1.16	0.76	0.447	0.41	0.04–4.03
Apgar scores at the 1st minute	1.5	2.08	0.72	0.472	4.48	0.08–265.05
Apgar scores at the 5th minutes	−3.59	2.71	1.33	0.185	0.03	0–5.59
Respiratory distress syndrome (RDS)	2.9	6.86	0.42	0.672	18.23	0–12653983.99
Bronchopulmonary dysplasia (BPD)	18.61	154044.9	0	1	121313114.3	0–∞
Transient tachypnea of the newborn (TTN)	4.34	7.03	0.62	0.537	76.85	0–74541129.34
Patent ductus arteriosus (PDA)	3.73	19.42	0.19	0.848	41.72	0–1402729651104787700
Intraventricular hemorrhage (IVH)	32.56	18875.53	0	0.999	138273450904040	0–∞
Neonatal pneumonia	4.91	3.87	1.27	0.204	136.03	0.07–267863.57
Hypoxic-ischemic encephalopathy (HIE)	−2.41	3.29	0.73	0.464	0.09	0–56.9
Neonatal depression	22.25	11058.25	0	0.998	4588890374.94	0–∞
Hemorrhagic disease of newborn (HDN)	20.53	11058.25	0	0.999	824843677.14	0–∞
Neonatal hyperbilirubinemia	−1.18	1.59	0.74	0.459	0.31	0.01–6.96
Disseminated intravascular coagulation (DIC)	10.67	5.16	2.07	0.038	43151.59	1.77–1054626464.67
Anemia of prematurity (AOP)	−15.2	132203.91	0	1	0	0–∞
Necrotizing enterocolitis (NEC)	−14.46	138368.14	0	1	0	0–∞
TORCH infections	−18.43	115735.57	0	1	0	0–∞
Neonatal seizures	0.57	191176.74	0	1	1.77	0–∞

## Data Availability

The data supporting the findings of this study can be provided by the corresponding author upon reasonable request.

## References

[1] Ohuma E. O., Moller A. B., Bradley E. (2023). National, Regional, and Global Estimates of Preterm Birth in 2020, With Trends From 2010: A Systematic Analysis. *Lancet*.

[2] Wu X. P., Gu C. L., Han S. P. (2021). A Multicenter Retrospective Study on Survival Rate and Complications of Very Preterm Infants. *Zhongguo Dang Dai Er Ke Za Zhi*.

[3] Lin L., Liu G., Li Y. (2022). Apgar Scores Correlate With Survival Rate at Discharge in Extremely Preterm Infants With Gestational Age of 25-27 Weeks. *Brazilian Journal of Medical and Biological Research*.

[4] Cnattingius S., Johansson S., Razaz N. (2020). Apgar Score and Risk of Neonatal Death Among Preterm Infants. *New England Journal of Medicine*.

[5] Zaigham M., Maršál K. (2020). Apgar Score in Premature Infants Associated With Neonatal Death Prediction. *Journal of Pediatrics*.

[6] Mitha A., Chen R., Altman M., Johansson S., Stephansson O., Bolk J. (2021). Neonatal Morbidities in Infants Born Late Preterm at 35-36 Weeks of Gestation: A Swedish Nationwide Population-Based Study. *Journal of Pediatrics*.

[7] Cao Y., Jiang S., Sun J. (2021). Assessment of Neonatal Intensive Care Unit Practices, Morbidity, and Mortality Among Very Preterm Infants in China. *JAMA Network Open*.

[8] Warnier H., Dauby J., De Halleux V. (2024). Prevention of Prematurity's Complications. *Revue Medicale de Liege*.

[9] Siffel C., Hirst A. K., Sarda S. P., Kuzniewicz M. W., Li D. K. (2022). The Clinical Burden of Extremely Preterm Birth in a Large Medical Records Database in the United States: Mortality and Survival Associated With Selected Complications. *Early Human Development*.

[10] Bell E. F., Hintz S. R., Hansen N. I. (2022). Mortality, In-Hospital Morbidity, Care Practices, and 2-Year Outcomes for Extremely Preterm Infants in the US, 2013–2018. *JAMA*.

[11] Kitaoka H., Konishi T., Shitara Y. (2024). Treatments and Outcomes of Neonatal Disseminated Intravascular Coagulation With and Without Neonatal Asphyxia: A Retrospective Study Using Nationwide Data in Japan. *Pediatrics and Neonatology*.

[12] Tana M., Tirone C., Aurilia C. (2023). Respiratory Management of the Preterm Infant: Supporting Evidence-Based Practice at the Bedside. *Children*.

[13] Boel L., Hixson T., Brown L., Sage J., Kotecha S., Chakraborty M. (2022). Non-invasive Respiratory Support in Preterm Infants. *Paediatric Respiratory Reviews*.

[14] Manley B. J., Cripps E., Dargaville P. A. (2024). Non-Invasive Versus Invasive Respiratory Support in Preterm Infants. *Seminars in Perinatology*.

[15] Bai R., Jiang S., Guo J. (2021). Variation of Neonatal Outcomes and Care Practices for Preterm Infants <34 Weeks' Gestation in Different Regions of China: A Cohort Study. *Frontiers in Pediatrics*.

[16] Davis R., Stuchlik P. M., Goodman D. C. (2023). The Relationship Between Regional Growth in Neonatal Intensive Care Capacity and Perinatal Risk. *Medical Care*.

[17] Gutvirtz G., Wainstock T., Sheiner E., Pariente G. (2022). Prematurity and Long-Term Respiratory Morbidity-What Is the Critical Gestational Age Threshold?. *Journal of Clinical Medicine*.

[18] Pollak M., Shapira M., Gatt D., Golan-Tripto I., Goldbart A., Hazan G. (2025). Transient Tachypnea of the Newborn and the Association With Preschool Asthma. *Annals of the American Thoracic Society*.

[19] Juul S. E., Wood T. R., Comstock B. A. (2022). Deaths in a Modern Cohort of Extremely Preterm Infants From the Preterm Erythropoietin Neuroprotection Trial. *JAMA Network Open*.

[20] van Beek P. E., Groenendaal F., Broeders L. (2021). Survival and Causes of Death in Extremely Preterm Infants in the Netherlands. *Archives of Disease in Childhood-Fetal and Neonatal Edition*.

[21] McDonald F. B., Dempsey E. M., O'Halloran K. D. (2020). The Impact of Preterm Adversity on Cardiorespiratory Function. *Experimental Physiology*.

[22] Syltern J., Ursin L., Solberg B., Støen R. (2022). Postponed Withholding: Balanced Decision-Making at the Margins of Viability. *American Journal of Bioethics: AJOB*.

[23] Ginglen J. G., Butki N. (2023). Necrotizing Enterocolitis. *Stat Pearls*.

[24] van der Heide M., Mebius M. J., Bos A. F. (2020). Hypoxic/Ischemic Hits Predispose to Necrotizing Enterocolitis in (Near) Term Infants With Congenital Heart Disease: A Case Control Study. *BMC Pediatrics*.

[25] Morgan A. S., Khoshnood B., Diguisto C. (2020). Intensity of Perinatal Care for Extremely Preterm Babies and Outcomes at a Higher Gestational Age: Evidence From the EPIPAGE-2 Cohort Study. *BMC Pediatrics*.

